# Oxytocin but not naturally occurring variation in caregiver touch associates with infant social orienting

**DOI:** 10.1002/dev.22290

**Published:** 2022-06-08

**Authors:** Alicja Brzozowska, Matthew R. Longo, Denis Mareschal, Frank Wiesemann, Teodora Gliga

**Affiliations:** ^1^ Department of Psychological Sciences Birkbeck, University of London London UK; ^2^ Department of Developmental and Educational Psychology University of Vienna Vienna Austria; ^3^ Baby Care Procter & Gamble Service GmbH Schwalbach am Taunus Germany; ^4^ Department of Psychology University of East Anglia Norwich UK

**Keywords:** infancy, oxytocin, parental care, social orienting, touch

## Abstract

Caregiver touch is crucial for infants’ healthy development, but its role in shaping infant cognition has been relatively understudied. In particular, despite strong premises to hypothesize its function in directing infant attention to social information, little empirical evidence exists on the topic. In this study, we investigated the associations between naturally occurring variation in caregiver touch and infant social attention in a group of 6‐ to 13‐month‐old infants (*n* = 71). Additionally, we measured infant salivary oxytocin as a possible mediator of the effects of touch on infant social attention. The hypothesized effects were investigated both short term, with respect to touch observed during parent–infant interactions in the lab, and long term, with respect to parent‐reported patterns of everyday touching behaviors. We did not find evidence that caregiver touch predicts infant social attention or salivary oxytocin levels, short term or long term. However, we found that salivary oxytocin predicted infant preferential attention to faces relative to nonsocial objects, measured in an eye‐tracking task. Our findings confirm the involvement of oxytocin in social orienting in infancy, but raise questions regarding the possible environmental factors influencing the infant oxytocin system.

## 1 INTRODUCTION

Parents spend considerable amounts of time in body contact with their infants, engaging in different types of tactile interactions (Bigelow & Williams, 2020; Hertenstein, 2002). While some interactions mediated by touch (e.g., feeding, securing the infant's position) are necessary for the infant's basic survival, generally the amounts and types of touch caregivers engage in go well beyond fulfilling these basic functions. Indeed, developmental psychology has recognized the function of touch in bonding (Norholt, 2020) and affective regulation (Fotopoulou et al., 2022) processes in infancy. Recently, we have seen an increase in scientific interest in the role that caregiver touch might play in promoting infant cognitive development in particular (Bales et al., 2018; Carozza & Leong, 2021; Cascio et al., 2018; Crucianelli & Filippetti, 2020; Gliga et al., 2019). Two possible mechanisms have been suggested to explain this alternative function of touch. One line of thinking, heavily influenced by research on tactile interaction in rodents, sees touch as an index of environment quality (Meaney, 2001). The presence of touch would indicate that the parent has the time and energy to engage in this costly means of interaction, implying that the environment is safe and full of resources, and therefore would be conducive to exploration and learning. This view is supported by the apparent stress‐buffering effects of touch (Morrison, 2016).

As a second possibility, touch might act as a social communicative cue, signaling the availability of the caregiver for social learning. According to this account, touch would therefore be akin to infant‐directed speech or direct eye gaze, enhancing the salience of social information (Akhtar & Gernsbacher, 2008; Della Longa et al., 2017; Peláez‐Nogueras et al., 1996; Wass et al., 2020).

Mutual gaze and infant‐directed speech have been shown to increase attention to the source of these signals, that is, the face (Cooper & Aslin, 1990; Farroni et al., 2002; Senju & Csibra, 2008). While there is rich evidence that touch is often used to get an infant's attention in the context of deafness or blindness (Bigelow, 2003; Koester et al., 2000), the communicative role of touch in typically developing infants has been rarely investigated. Simpson et al. (2019) demonstrated that after an interaction with a caregiver that did not involve any touch but did involve mutual gaze, 1‐week‐old macaque monkeys showed a preference for a nonsocial video (plastic bag floating in the wind) rather than a social video (conspecific producing an affiliative/positive facial expression), when presented with both of them, side by side, and at the same time. However, if the interaction with the caregiver included stroking, monkeys’ preferences were shifted such that they attended equally to both types of videos. In human infants, when the presentation of a face with averted gaze was accompanied with gentle stroking, the infants later recognized the identity of the face, but not when the face was initially accompanied by brush tapping or presented without concurrent tactile stimulation (Della Longa et al., 2017). However, it is unclear if this effect reflects increased attention to faces in response to touch, as in the study by Della Longa et al. (2017) and in two subsequent studies no effects of touch on looking times to faces were found (Della Longa et al., 2020; Nava et al., 2020); thus, it is possible that a more general attention effect compatible with touch decreasing stress responses and promoting learning was involved.

Nevertheless, in these studies (Della Longa et al., 2020, 2017; Nava et al., 2020), the infants were presented with only one face stimulus, or two face stimuli side by side, both in their visual field. This may have led to a ceiling effect in which infants’ interest in the face stimulus (present with a lack of competing stimuli to look at) was already very high. When measuring social preference, a more complex scene in which nonsocial stimuli are presented alongside faces could be a more sensitive and consequently more appropriate approach. Importantly, social preference measured in such a way has recently been shown to predict later language development (Portugal et al., 2022). Indeed, in 4‐ to 6‐year‐old children, naturally occurring variation in caregiver touching patterns (as observed during an interactive play session) correlated with attention to social stimuli. Those children who were touched more frequently by their mothers during the play session were more distracted by faces (relative to nonsocial stimuli – houses) in an object categorization task; that is, when performing an object‐categorization task where the target stimuli were overlaid on pictures of faces or houses, their responses were on average less accurate with face pictures (vs. house pictures) in the background (Reece et al., 2016). Moreover, the frequency of maternal touch during a play session has been shown to predict activation of and connectivity between areas belonging to the “social brain,” including right posterior superior temporal sulcus and left insula, in 5‐year‐olds (Brauer et al., 2016). Additionally, there is indirect evidence that touch might increase orienting to social stimuli in infancy, as social orienting was shown to be predicted by salivary oxytocin levels (Nishizato et al., 2017), which increase in response to kangaroo care in infants born prematurely (Vittner et al., 2017).

Oxytocin is a neuromodulator and hormone, synthesized in the paraventricular nucleus and supraoptic nucleus of the hypothalamus (Walum & Young, 2018). Although commonly referred to as a “love hormone,” most scientists would agree that rather than being a special bonding or nurturing molecule, oxytocin acts more generally, through modulating the salience and reinforcing nature of social stimuli (Quintana et al., 2019; Young, 2013). Our understanding of the role of oxytocin is largely based on animal research, but numerous studies have demonstrated its involvement in social cognition and attention in human adults (Guastella et al., 2008; Hovey et al., 2020; Tillman et al., 2019). Much of what we know about the causal role of oxytocin in behaviour comes from studies involving intranasal administration of the hormone. In adults, intranasal administration of oxytocin was found to increase gaze to the eye region of human faces (Guastella et al., 2008) and to bias attention toward faces relative to houses (Hovey et al., 2020). An event‐related potential study showed that intranasally administered oxytocin affects early stages of face processing, further supporting the notion of its role in modulating the salience of socially informative cues (Tillman et al., 2019). Given that intranasal administration of oxytocin is not commonly used with developmental populations and in particular, no studies to date have used this method with infants, developmental research examining short‐term oxytocin effects relies to a large extent on studies involving measuring oxytocin in blood, urine, or saliva. Accordingly, salivary oxytocin was found to correlate positively with attention to mouth and eye regions in infants aged 5 months up to children aged 7.5 years (Nishizato et al., 2017). In 4‐month‐olds, salivary oxytocin was shown to be positively correlated with gazes at mother during a naturalistic interaction, but only in infants whose mothers exhibited high affect attunement (defined as maintaining attention and warm sensitivity; Markova & Siposova, 2019).

Thus, it is possible that caregiver touch would bias infant attention toward social stimuli through the release of oxytocin (Walker et al., 2017), consistent with the hypothesis of touch as a signal to orient to and learn from the caregiver. Yet studies on the associations between caregiver touch and infant oxytocin activity have provided inconsistent results. For instance, Kommers et al. (2018) reported a decrease in salivary oxytocin levels in premature infants during kangaroo care. What is more, naturally occurring variation in caregiver touch in the postnatal period was not associated with DNA methylation at the oxytocin receptor gene when the child was 4–5 years old (Moore et al., 2017), which calls into question the hypothesis of oxytocin‐mediated long‐term consequences of caregiver touch on development in humans, previously demonstrated in rodents (Francis et al., 2000). Thus, although there are compelling reasons to hypothesize the oxytocin‐mediated effects of touch on social attention in infancy, the evidence is currently lacking.

In the present study, we aimed to measure the associations between naturally occurring variation in caregiver touch, infant salivary oxytocin, and social attention. Specifically, by naturally occurring variation in caregiver touch we mean touch‐related behaviors that caregivers spontaneously engage in, as opposed to touch introduced in the form of an intervention (e.g., kangaroo care; see, e.g., Hardin et al., 2020), or touch applied in highly controlled experimental settings (e.g., stroking at a predefined velocity; see, e.g., Aguirre et al., 2019), given that animal literature points to strong developmental impact of such behaviors. We tested the hypothesis that touch acts as a social communicative cue by asking whether it increases oxytocin levels as well as social attention. We captured social attention by measuring infant attention to faces in an eye‐tracking task where complex scenes consisting of faces alongside several nonsocial objects were presented to the infant (Gliga et al., 2009). Additionally, we tested the association between infant oxytocin and social attention.

The reviewed research has focused both on immediate and long‐term effects of touch. Therefore, in this study we capture parental touch both short term, by measuring touch during parent–child interaction during the visit at the lab, and long term, as assessed with self‐report questionnaires. We therefore first asked whether more self‐reported touch from the caregivers, representing more touch received on a daily basis, would predict higher levels of oxytocin upon arrival to the lab, as well as more looking to the face relative to nonsocial objects. We also hypothesized that more touch received from the caregiver during an interaction in the lab would be associated with a larger increase in oxytocin from before to after the interaction, as well as more looking at the face in the eye‐tracking task, representing the short‐term effects of parental touch. Additionally, we predicted that higher levels of oxytocin would be associated with more looking at the face.

Finally, while the majority of research on touch in infancy has been focused roughly on the first 6 months of life, we wanted to extend the age span studied to see if the putative effects of caregiver touch would also be observed later in infancy (especially considering the evidence that these effects might be present in early childhood; Reece et al., 2016). Specifically, we included infants aged between 6 and 13 months, recruited into two age groups, 6‐ to 8‐month‐olds, who typically spend a lot of time in close physical proximity to the caregiver, and 11‐ to 13‐month‐olds, who are capable of a larger degree of motor independence, and can therefore move farther away from the caregiver and rely more on communicative cues other than touch. As the data presented here were collected as a part of a larger study, the inclusion of these age groups was also motivated by age‐related hypotheses pertaining to other collected measures.

## 2 METHODS

### 2.1 Participants

The study was conducted at the Baby Care Research & Development Centre (Procter & Gamble, Schwalbach am Taunus, Germany). Seventy‐one caregiver–infant dyads were recruited from a database of families living in the Taunus and Frankfurt am Main area interested in research taking place at the Baby Care Research & Development Centre. The infants were recruited into two age groups: 6‐ to 8‐month‐olds (*n* = 39, *M* = 7 months 21 days, 21 males and 18 females) and 11‐ to 13‐month‐olds (*n* = 32, *M* = 12 months 10 days, 17 males and 15 females). Sixty‐nine of the primary caregivers were female, and the remaining two were male. Inclusion criteria for the study were as follows: infant gestational age at the time of birth >37 weeks, no diagnosed developmental disorders, and German fluency (caregiver). Of note, we have previously reported analyses of the associations between different measures of caregiver touch in these participants (Brzozowska et al., 2021). The present study was conducted according to guidelines laid down in the Declaration of Helsinki, with written informed consent obtained from a parent or guardian for each child before any assessment or data collection. All procedures involving human subjects in this study were approved by the Research Ethics Committee at the Department of Psychological Sciences, Birkbeck, University of London.

### 2.2 Measures

#### 2.2.1 Caregiver touch

##### Parent–Infant Caregiving Touch Scale

We used an adapted version of the Parent–Infant Caregiving Touch Scale (PICTS; Koukounari et al., 2015) as a self‐report measure of caregiver touch. Four items of the scale refer to stroking of different body parts, while the rest pertain to other forms of touch and communication: picking up, cuddling, rocking, kissing, holding, talking to, watching, and leaving the baby to lie down. Caregivers indicate how often they engage in those behaviors by picking a level on a 5‐point Likert scale ranging from 1 = *Never* to 5 = *A Lot*. The scale was translated into German. In addition to the original items, we added two extra items: (i) *I sleep in the same bed with my bab*y, and (ii) *I carry my baby in a sling*. We computed a total score (PICTS score), composed of all items in the questionnaire, in order to obtain a general measure of touching behaviors (Brzozowska et al., 2021). The Cronbach's *α* value for the total score in our sample was .71, indicating appropriate internal consistency (Field et al., 2012).

##### Parent–child interaction‐coded touch

Interactions between parents and their children were filmed and later coded for parental touch patterns. Parent–child interaction (PCI) was observed in two situations: (i) 10 min of free play (PCI‐FP) and (ii) 10 min during which the parent orally answered questions (PCI‐Q) from the Infant Behaviour Questionnaire—Very Short Version (IBQ‐R; Putnam et al., 2014). We have previously found that while there is a good degree of agreement between caregiver self‐reported and observed touch, including a PCI condition in which the parent is engaged in another primary activity while interacting with their infant provides additional information about the variation in caregiving behaviors that the other measures do not capture (Brzozowska et al., 2021).

The PCI videos were later coded offline frame by frame using Datavyu software (Datavyu Team, 2014) at 30 frames per second. For both conditions, PCI‐FP and PCI‐Q, 5 min of interaction were coded, from the third to the seventh minute of the interaction in each condition. The total duration of overall touch (i.e., any time the infant was being touched at all during the 5 min of interaction being coded) was computed in both PCI conditions (Brzozowska et al., 2021). Interrater reliabilities were calculated on 20% of interactions using a two‐way mixed, consistency, single‐measures intraclass correlation (Hallgren, 2012; McGraw & Wong, 1996). The secondary coder did not have access to these scores at all. For the total duration of touch, the ICC was .92, indicating excellent reliability (Cicchetti, 1994).

The resulting measure used in the analyses, Observed Touch, was a sum of the duration of touch in PCI‐FP and PCI‐Q, measured in seconds.

#### 2.2.2 Oxytocin

While some concerns have been raised around the reliability of the salivary oxytocin measure (Uvnäs‐Moberg et al., 2020), salivary oxytocin detected with enzyme immunoassay kits has been shown to be a reliable biomarker in adults, capturing reproducible changes associated with lactation and massage (Carter et al., 2007). Although comprehensive reliability assessments in developmental populations are lacking (likely because of the difficulty associated with obtaining multiple saliva samples, particularly from infants), salivary oxytocin has been shown to correlate meaningfully with various behavioural and physiological variables in adults and children (Uvnäs‐Moberg et al., 2020). Critically, research showing effects of kangaroo care on oxytocin in infants (Vittner et al., 2017), as well as associations between oxytocin and social attention in infants and children (Nishizato et al., 2017), has used salivary oxytocin detected with enzyme immunoassay kits (by Enzo Life Science). Given the significance of these findings to our study, as well as the relative noninvasiveness of saliva sampling procedure with infants, we decided to adopt a similar approach.

Infant saliva samples were obtained using Salivette^®^ (Sarstedt, Rommelsdorf, Germany). The parents were asked not to feed their children for 45 min prior to their arrival to the lab. Samples were collected at the beginning of the dyad's visit in the lab, shortly after acquainting them with the lab, and after an approximately 40‐min period of parent–infant interaction, resulting in a maximum of two samples per infant. At each time, parents were asked to put on a glove and put the Salivette^®^ in their child's mouth for them to chew for 1 min until it was saturated with saliva (see Nishizato et al., 2017). During saliva collection, the caregivers could position the infant however they wanted to make the saliva collection procedure as comfortable for the infant as possible. Throughout this procedure, the experimenter blew bubbles to entertain and distract the infant.

Saliva samples were stored at −20°C until assay. A commercially available kit (Oxytocin EIA kit, ADI‐901‐153, Enzo Life Science) was used to determine the concentration of oxytocin (OT). The limit for detection of the assay was 8.3 pg/ml (this is comparable with previous studies; e.g., Markova & Siposova, 2019; White‐Traut et al., 2009). Saliva was recovered from the swabs by centrifugation. The assay procedure meticulously followed the kit's instructions (and was comparable with, e.g., Huffmeijer et al., 2012; Markova & Siposova, 2019) and was performed by a trained technician at Procter & Gamble.

Four different measures of oxytocin activity were used in this study:

1. OT1: salivary oxytocin at timepoint 1, at the beginning of the visit, representing infant's baseline oxytocin level;
2. OT2: salivary oxytocin at timepoint 2, after ∼40 min of parent–child interaction, likely representing infant's oxytocin level in response to interaction with the caregiver;
3. OT AUC: area under the curve with respect to ground (i.e., zero), a widely used index of increase/decrease in oxytocin level that incorporates information about time distance between the measurements (Pruessner et al., 2003); here (following, e.g., Markova & Siposova, 2019) used as an index of infant total hormonal output and analyzed with regard to long‐term parental touch;
4. OT2 – OT1: the difference between OT1 and OT2, an index of change in oxytocin levels from before to after the interaction with the caregiver; here used as a measure sensitive to changes occurring within the session (see, e.g., Vittner et al., 2017) and analyzed with regard to short‐term parental touch.

#### 2.2.3 Infant social attention—Face Pop Out task

**FIGURE 1:**
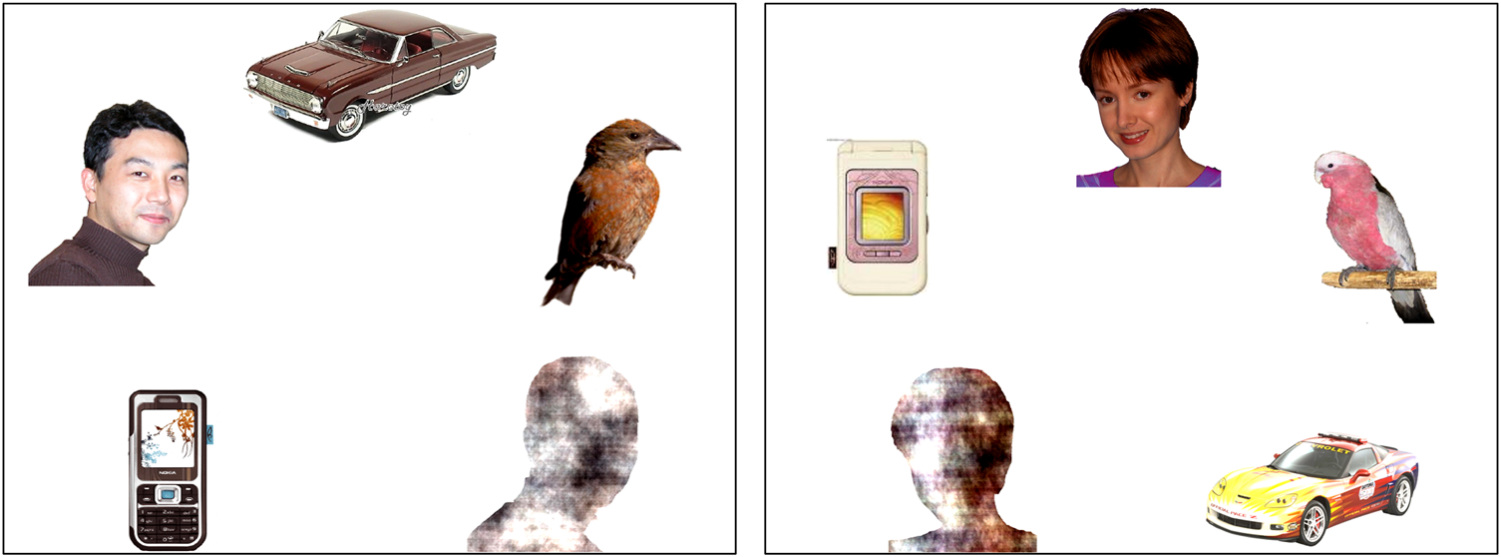
Example slides from the Face Pop Out task

In this task, infants were presented with a complex visual array containing faces among five other visual objects, such as cars, phones (nonsocial everyday objects), and scrambled face stimuli (matched with the faces for low‐level psychophysical properties such as frequency content, color distribution, and outer contour; for more information about the types of pictures used, see Elsabbagh et al., 2013; Gliga et al., 2009; Halit et al., 2004). Example slides are shown in Figure 1. The infants were presented with seven different slides, for 10 s each. Before each slide, a small attention‐grabber stimulus was presented in the center of the screen to ensure that the infant's gaze was directed to the center. To maintain the infant's attention, the visual presentation was accompanied by unrelated music. Their gaze was recorded with a 120‐Hz Tobii x120 eye tracker.

This task has been used in various studies, with measures such as proportion of first looks to the face (Gliga et al., 2009) and peak look durations to the face (Gui et al., 2020; Hendry et al., 2018) being extracted. In this study, we used it to assess infants’ interest in faces as compared to nonsocial stimuli, to verify whether touch and related measures of arousal can affect the distribution of attention. Thus, the measure of interest was the proportion of time the infants spent looking at the face stimulus (i.e., infant's gaze was within a rectangular area of interest around the face) with respect to the total time they spent looking at a slide (de Klerk et al., 2014; Elsabbagh et al., 2013; Portugal et al., 2022; Telford et al., 2016). This proportion was computed for each slide where the infant's gaze was on the screen for at least 6.7 s of the time of its presentation (67% of the time; analogous to de Klerk et al., 2014; Elsabbagh et al., 2013). Considering the strong attention‐grabbing properties of faces (Gliga et al., 2009), we wanted to include only those trials in which infants looked at the screen long enough for significant variability to occur, in order to avoid a ceiling effect. The proportions were averaged from between one and seven slides per infant (depending on how many valid trials an infant provided) to provide a more stable characterization of individual differences.

### 2.3 Procedure

The data presented here were collected as a part of a larger study investigating the relationship between caregiver touch and infant developmental outcomes, some results of which we already published (Brzozowska et al., 2021). Other measures such as salivary cortisol, heart rate, and infant performance in table top and eye‐tracking tasks measuring exploratory behaviour and attention were also taken. Here, we provide a brief description of the procedure in order to inform about the timing of the experimental steps (Brzozowska et al., 2021).

The caregiver–infant dyads were welcomed to the laboratory and provided informed consent before the start of the study. The caregivers were notified that their behaviour during the entire duration of the visit would be video‐recorded (unless they withdrew their consent), but we did not tell them that we were specifically interested in touching behaviors until after the study. When the participants had familiarized themselves with the setting, saliva samples were taken from the infant by the caregiver using Salivette^®^ Cotton Swabs (Sarstedt, Rommelsdorf, Germany). After about 7 min of activities associated with heart rate measurements, the parent was informed that starting from now, everything happening in the room would be filmed until the experimenter said otherwise. Next, the parent was asked to change the baby's diaper and, when they were done, Parent–Child Interactions, Free Play (PCI‐FP), and Questions (PCI‐Q) began. Both interactions took place in the same room, one after the other. As we wanted to create a setting where potential caregiver touch would be maximized, there were no toys in the room, only a blanket, a beanbag, and two cushions. For PCI‐FP, parents were asked to play with their children like they normally would at home, without any toys, and to try to remain close to the area marked out by the blanket, for the cameras to be able to capture the interaction. The experimenter was not present in the room, but observed the free play through a one‐way mirror in an adjacent room (a fact the caregivers knew about).

**FIGURE 2:**
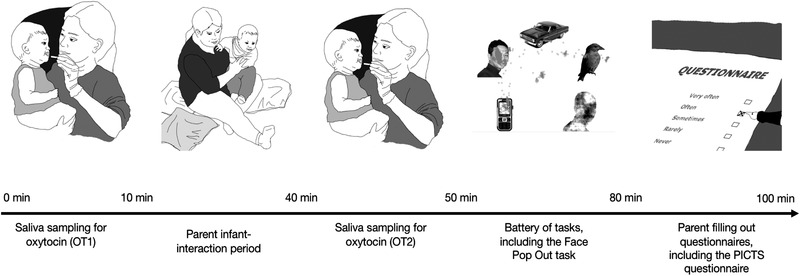
Approximate time course of the dyad's visit in the lab

The PCI‐FP part of the study started after 10 min of free play: the experimenter returned to the main room, sat down on the blanket, and asked questions from the IBQ‐R for another 10 min. Afterward, saliva samples were collected again, and the infant then participated in the table top and eye‐tracking tasks (including the Face Pop Out task, as well as tasks not relevant to the present investigation). At the end of the visit, the parent filled in the Parent–Infant Caregiving Scale and another questionnaire (Social Touch Questionnaire; Wilhelm et al., 2001). The entire visit in the lab lasted on average between 1.5 and 2 h. An approximate time course of the visit is depicted in Figure 2.

### 2.4 Analytical approach

Missing data are a pervasive problem in developmental research, and our dataset was not exempt from it (see Section 3.1 for details). We used multiple imputation to create and analyze 20 multiply imputed datasets, following the guidance described by Graham et al. (2007). Multiple imputation is often considered a preferred way of dealing with missing data, as it improves accuracy and statistical power relative to other missing data techniques (Enders, 2013; Little et al., 2016). Incomplete variables were imputed under fully conditional specification, using the default settings of the *mice* 3.0 package (van Buuren & Groothuis‐Oudshoorn, 2011). Multiple regression models (forced entry) were fitted to the data to test the hypothesized predictions. The parameters of substantive interest were estimated in each of the 20 imputed datasets separately, and combined using Rubin's rules (Rubin, 1987). All the reported results come from the imputed datasets; for comparison, the analyses performed on the subset of pairwise‐deleted complete cases are reported in the Supporting Information.

Eye‐tracking data were analyzed using a custom script written in MATLAB R2017a (Mathworks, Natick, MA). Look target coordinates were calculated from an average of *x* and *y* gaze locations from both eyes; single‐eye data points were used where data from one eye were missing. Periods of data loss (due to blinks or temporary inaccuracy of data capture) up to 150 ms were linearly interpolated. The statistical analyses were performed in R (version 3.6.0.; R Core Team, 2019).

## 3 RESULTS

### 3.1 Descriptive statistics

Sixty‐eight caregivers provided the PICTS scores, with data from three participants missing due to caregivers not completing the questionnaire (two participants) or experimenter error (one participant). Observed Touch data were available for 68 infants, with data from three participants missing due to technical problems with the video recordings.

There was a substantial amount of missing data for OT1 (44%) and OT2 (39%) due to insufficient volume of saliva collected (and, in some cases, possibly an error in computing OT, i.e., concentrations below the limit of detection). Some infants refused to have the Salivette® cotton swabs put in their mouths at all, and a number of them did not suck on the swabs long enough to provide enough saliva. Nevertheless, the amount of missing data is comparable to that in similar previous studies, such as Markova and Siposova (2019) who reported 30%–50% missing OT values in their study with 4‐month‐olds. Additionally, four OT2 values were removed due to the mothers feeding their children during the period of interaction between collection of the samples. Group means and differences in variables of interest between the infants who did and did not provide OT data points are shown in Table S1. Among the participants who had both OT1 and OT2 data points, the correlation between the two measures was *r*(31) = .37, *p* = .034, speaking for the reliability of the measure in our study.

Sixty‐four infants contributed Face Pop Out Scores, with data from seven participants missing because they did not participate in the eye‐tracking session at all due to fussiness. Infants contributed data from an average of 4.4 slides (*SD* = 1.9): 4.6 (*SD* = 2.1) in the 6‐ to 8‐month‐olds group, and 4.2 (*SD* = 1.7) in the 11‐ to 13‐month‐olds group.

Detailed descriptive statistics for the measures used in the subsequent analyses (original data) are reported in Table 1.

**TABLE 1:** Descriptive statistics for infant age in days, PICTS scores, Observed Touch, OT1, OT2, OT2 – OT1, OT AUC, and Face Pop Out Scores, split by age group

		Age (days)	PICTS	Observed touch (s)	OT1 (pg/ml)	OT2 (pg/ml)	OT2 – OT1 (pg/ml)	OT AUC (pg·min/ml)	Face Pop Out Score
6–8‐month‐olds	Mean (*SD*)	232 (30)	55 (5)	325 (162)	102 (56)	117 (75)	26 (70)	4991 (2390)	0.50 (0.17)
	Min–max	170–272	43–65	67–600	8–231	20–288	−79 to 144	1766–9360	0.16–0.96
	*N*	39	38	37	24	25	20	20	33
11–13‐month‐olds	Mean (*SD*)	371 (30)	54 (6)	203 (110)	103 (61)	145 (89)	27 (92)	5160 (2004)	0.46 (0.14)
	Min–max	335–420	39–65	73–503	25–198	28–291	−93 to 184	2417–8464	0.12–0.72
	*N*	32	30	31	16	15	11	11	31

### 3.2 Does caregiver touch predict infant oxytocin levels?

We found that infant oxytocin levels upon arrival to the lab were not predicted by the amount of touch reported by the caregivers: in the model predicting infant OT1 with infant age group and the PICTS score, neither age (*β* = –0.08, *SE* = 0.32, *t* = −0.25, *p* = .80) nor the PICTS (*β* = 0.01, *SE* = 0.13, *t* = 0.07, *p* = .94) score was a significant predictor.

We also did not observe the hypothesized short‐term effects of caregiver touch on the change in infant oxytocin levels (OT2 – OT1): neither age group (*β* = 0.08, *SE* = 0.32, *t* = 0.24, *p* = .81) nor Observed Touch (*β* = −0.06, *SE* = 0.14, *t* = −0.40, *p* = .69) was a significant predictor of change in the oxytocin levels in the infant.

### 3.3 Does caregiver touch predict infant social attention?

Neither age group (*β* = –0.27, *SE* = 0.24, *t* = −1.12, *p* = .27) nor the PICTS score (*β* = –0.09, *SE* = 0.12, *t* = −0.73, *p* = .47) significantly predicted infant Face Pop Out scores, indicating no evidence of long‐term effects of caregiver touch on infant social attention.

We did not find evidence of the putative short‐term effects of touch on infant social attention, as neither age group (*β* = −0.33, SE = 0.26, t = −1.27, p = 0.21) nor Observed Touch (*β* = −0.09, *SE* = 0.14, *t* = −0.68, *p* = .50) significantly predicted infant Face Pop Out scores.

### 3.4 Does oxytocin predict infant social attention?

**FIGURE 3:**
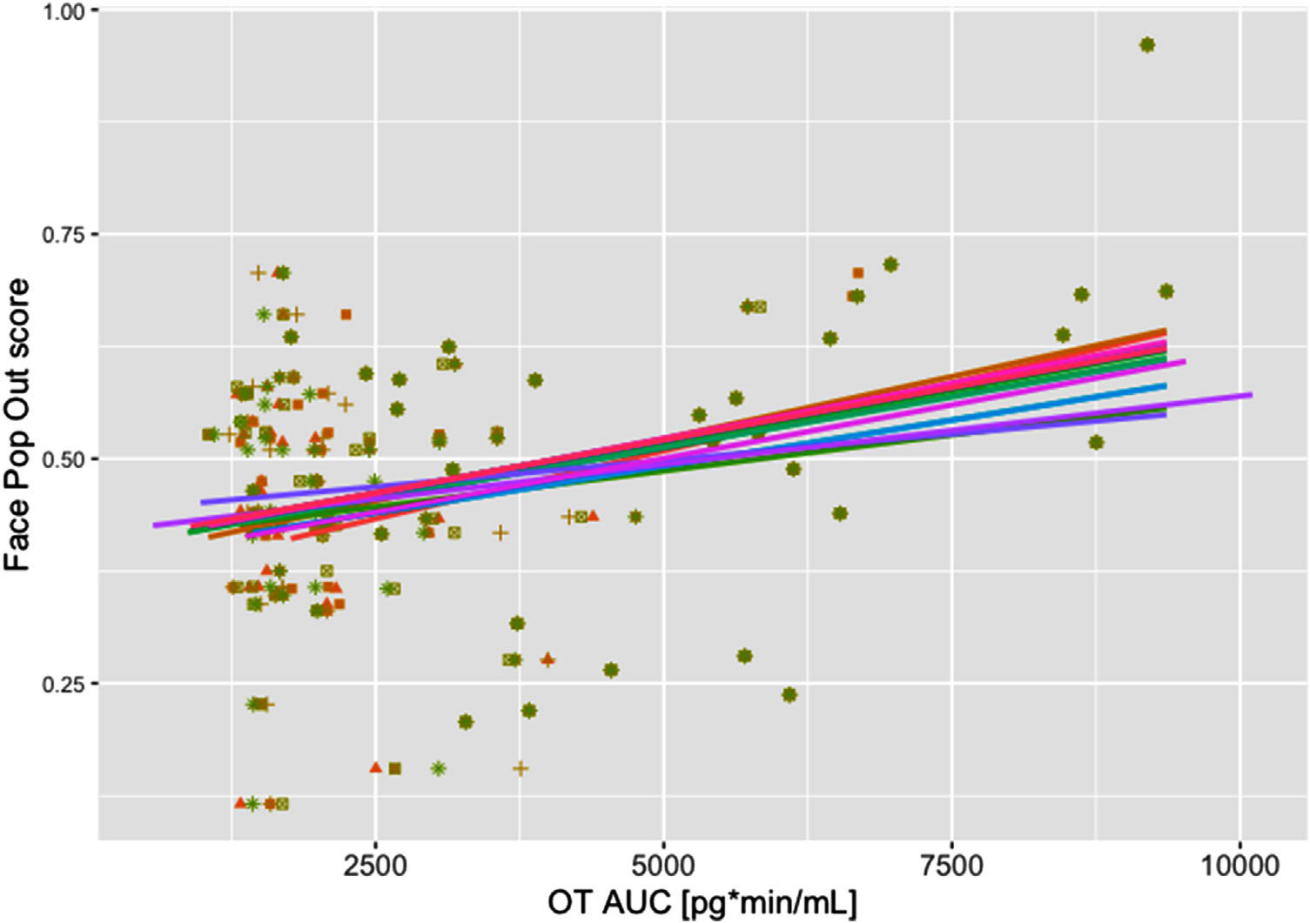
Scatterplot showing Face Pop Out Scores against infant OT AUC (pg·min/ml). Different shapes of data points correspond to the 20 datasets generated by multiple imputation. Different colors of the fitted regression lines correspond to separate linear regressions fitted to the 20 imputed datasets.

A regression model predicting infant social attention with oxytocin AUC revealed that OT AUC (*β* = 0.32, *SE* = 0.14, *t* = 2.34, *p* = .02) significantly predicted infant Face Pop Out score. The higher the values of infant OT AUC, the longer the infants looked at the face relative to the other objects (see Figure 3).

### 3.5 Additional analyses

Given that we did not find evidence for associations between touch and infant oxytocin or social attention, we did not conduct further mediation analyses. Instead, we performed an exploratory investigation into the correlations between the types of touch most commonly used during parent–infant interactions (hugging/holding, stroking/caressing, kissing/patting, moving limbs or body, static touch; for a detailed coding scheme, see Brzozowska et al., 2021) and change in infant oxytocin levels and Face Pop Out scores. No significant associations between the measures were found; detailed analyses are reported in Table S2. We also conducted additional coding of proximity during PCI‐FP (following Krol, Moulder, et al., 2019), and report the analyses using this measure in the Supporting Information (Section 4). In the Supporting Information (Section 5), we also report and comment on the associations between infant age and Face Pop Out scores.

## 4 DISCUSSION

Our study aimed to investigate the associations between naturally occurring variation in caregiver touch and infant oxytocin levels, as well as overt attention to faces. We hypothesized a mediation model, whereby infants receiving more touch from the caregivers would exhibit longer looking times to faces, relative to nonsocial stimuli, and that this association would be mediated by an increase in infant salivary oxytocin levels. If found, this association would support the notion that a way in which caregiver touch promotes cognitive development in infancy is through its oxytocin‐mediated effects on social orienting. This hypothesized model was tested both long term (the associations with everyday touch, as reported by the caregiver) and short term (the associations with touch observed during the dyad's visit in the lab).

We found no evidence for either of the hypothesized effects of touch on infant social attention. Thus, our study adds to the number of studies that found no associations between either experimentally applied stroking (Della Longa et al., 2020, 2017; Nava et al., 2020) or naturally occurring variation in caregiver touch (Tanaka et al., 2021) and infant overt attention to faces. The way social orienting was measured in our study, with multiple nonsocial objects competing with the face for infants’ attention, possibly captured infant attentional bias toward social stimuli better than the measures used in previous studies. Yet, the picture emerging from the research so far is that no measures of social attention based on looking times associate with measures of tactile stimulation provided to the infant.

Despite the seeming lack of effects of touch, we did find evidence that oxytocin predicts social attention in 6‐ to 13‐month‐olds. While several studies have reported similar effects in adults (Ellenbogen, 2018; Guastella et al., 2008; Hovey et al., 2020) and children (Fujisawa et al., 2014; Suzuki et al., 2020), to our knowledge, only one study to date (Nishizato et al., 2017) reported this in infants. Nishizato et al. (2017) showed a positive correlation between salivary oxytocin and fixation time spent on the eye area of the face in infants and children aged 5 months to 7.5 years, indicating the involvement of oxytocin in attention to socially salient stimuli early in development. While both attention toward the eye area and attention to faces relative to other objects (in the Face Pop Out task—the same task we used in our study) are impaired in infants born prematurely (Telford et al., 2016), it is unclear to which extent these deficits share a common mechanism. Our study further extends Nishizato et al.’s (2017) finding to the cases in which the infant's attention is distributed between faces and nonsocial stimuli, and implicates oxytocin as an important driver of these different aspects of social attention in infancy.

If oxytocin supports social attention in infancy, it is vital to identify which factors affect the oxytocin system. The measure we used to predict social orienting in infants, oxytocin AUC with respect to ground, indexes infant “total hormonal output” in terms of both intensity as well as sensitivity (Khoury et al., 2015; Pruessner et al., 2003). Short‐term fluctuations in infant oxytocin have been linked to time spent playing social games with the mother (Markova, 2018) and kangaroo care (Vittner et al., 2017); the latter has also been linked to long‐term changes in oxytocin levels over a period of 3 months (Hardin et al., 2020). Because few studies on oxytocin in infants have been published to date, we particularly lack knowledge about the time scales at which different factors could affect infant oxytocin levels (i.e., what could affect the baseline, as well as the change component of infant oxytocin levels).

The fact that we did not observe associations between caregiver touch and infant oxytocin levels might suggest that contrary to our hypotheses, caregiver touch is not a significant factor in shaping infant social attention. Yet, given the reports of kangaroo care increasing oxytocin in preterm (Vittner et al., 2017) and full‐term (Hardin et al., 2020) infants, it is also possible that the associations between touch and infant oxytocin were not captured due to insufficient amounts or types of touch occurring in our study. For instance, holding, the type of touch most closely resembling kangaroo care, was used by parents for about 25% of the time on average in the interactions we observed in the lab (Brzozowska et al., 2021), while the effects of kangaroo care have been observed for continuous periods of stimulation lasting about an hour (Hardin et al., 2020; Vittner et al., 2017). It is possible that one of the main variables of interest in our study, the total duration of touch, was simply not a sensitive enough measure of parent–infant touch interactions. However, further exploratory analyses (reported in the Supporting Information) using different touch measures did not reveal any significant patterns of associations between the different types of touch and infant oxytocin and social attention.

Furthermore, it is also possible that social attention in infancy is not subject to much environmental influence (Constantino et al., 2017; Portugal et al., 2022). Constantino et al. (2017) found that the way infants view social scenes is strongly influenced by genetic factors. In particular, preferential attending to eye and mouth regions of the face was the most heritable of the social attention characteristics measured in their study (Constantino et al., 2017), a finding consistent with what was later reported with regard to attention to faces relative to nonsocial objects by Portugal et al. (2022). Although the exact genes involved were not investigated in these studies, previous research demonstrated that the oxytocin receptor gene (OXTR) is involved in modulating infant neural response to emotional faces (Krol, Puglia, et al., 2019). Thus, it follows that the methylation of the OXTR gene would affect certain aspects of infant social information processing. However, no associations between OXTR methylation and naturally occurring variation in caregiver touch have been found (Moore et al., 2017).

Conversely, in a longitudinal study, Krol, Moulder, et al. (2019) found that OXTR gene methylation reduction at 18 months was predicted by higher maternal engagement (here defined as a combination of maternal proximity, talkativeness, and attention during a free‐play interaction) at 5 months. Although the authors also coded maternal touches, they found that the touches occurred relatively rarely and thus dropped them from the analyses. Thus, it may be that proximity, an important aspect of parent–infant interaction (Barnett et al., 2021), rather than physical touch, exerts influence over infants’ oxytocin system. Inspired by these results, we also looked into caregiver–infant proximity during free play (analyses reported in the Supporting Information), but found no evidence of associations between proximity and oxytocin or social orienting. Perhaps, as in Krol and colleagues’ (2019) study, these effects are observable at longer timescales.

Alternatively, a level of parental attunement or interactional synchrony might be needed for touch to affect infant hormonal response (and, consequently, social attention) (Feldman et al., 2010). Although the animal work (Caldji et al., 1998; D'Amato et al., 1998; Liu et al., 1997) shows effects of the sheer amounts of tactile stimulation provided on exploratory behaviors, it is likely that in human infants the degree to which parental touch is responsive to the infant's needs in given circumstances plays an important role. Indeed, it has been argued that synchrony between interactional partners—in particular, within a caregiver–infant dyad—plays a crucial role in the various neurobehavioral outcomes of the interaction (Markova et al., 2019; Schirmer et al., 2021). Interestingly, Crucianelli et al. (2019) showed that the social cognitive ability to understand an infant's mental state (called maternal mind‐mindedness and coded from parent–infant interaction videos) was predictive of the amount of touch that was noncontingent with infants’ emotional state (i.e., higher mind‐mindedness resulted in lower levels of noncontingent touch), but was not predictive of the emotion‐contingent touch. This finding suggests that the nonattuned touches might constitute an especially meaningful part of the variation in caregiver touch, potentially confounding any analyses focused on sheer amounts of tactile stimulation. More generally, the main insight coming from the research on synchrony and attunement is that the impact of caregiving behaviour is dependent on infant state, something that future research should investigate in more detail with respect to touch.

Recent years have also brought insights into the neural mechanisms underlying the impact of parental touch on infant development. Mateus et al. (2021) demonstrated that 7‐month‐olds whose mothers exhibited lower maternal sensitivity showed stronger neural activation in the left somatosensory cortex and right temporal cortex (as measured with oxy‐hemoglobin concentrations using functional near‐infrared spectroscopy) in response to affective touch, likely mediated by exposure to maternal touch. This finding adds to those from previous studies showing that infants’ processing of touch is shaped by their past experiences (Addabbo et al., 2021; Aguirre et al., 2019). Additionally, recent research has demonstrated that the effects of affectionate caregiver touch durations occurring during free play observed in the lab on mother–infant synchrony were observable at a neural but not physiological level in mothers and their 4‐ to 6‐month‐olds (Nguyen et al., 2021); it might be the case that the communicative role of touch in infancy is not mediated by arousal regulation or hormonal response. Although some studies reveal that cortical specialization to stroking might not develop until the end of the first year of life (Miguel et al., 2017; Pirazzoli et al., 2018), more studies examining infant brain activity in response to various types of caregiver touch, ideally combined with hormonal measures, could help us better understand the mechanisms involved.

We must acknowledge several limitations of our study. First, we largely drew inspiration from animal work demonstrating the consequences of naturally occurring variation in caregiver tactile behaviors on the exploratory behaviour of the offspring (Caldji et al., 1998; D'Amato et al., 1998; Liu et al., 1997). Yet, naturally occurring variation in caregiver touch captured in studies relying on voluntary recruitment and self‐report or short observation in the lab most likely does not capture the entire spectrum of caregiver behaviors. Even though in our study we observed significant variability in parental touch (as indexed for instance by the distribution of the PICTS scores), it is probable that the measures we used were not sensitive to the extreme ends of the caregiver behaviour spectrum. Emerging technologies, such as devices recording body contact (Yao et al., 2019), could partially address this issue by allowing us to capture touching behaviors over extended periods of time and in infants’ natural environment, and thus might be the future of touch research in infancy. Moreover, one of our main measures of interest, salivary oxytocin, has been associated with some controversies about its validity and specificity (Uvnäs‐Moberg et al., 2020), and it has also yielded a substantial amount of missing data in our study. Accordingly, our results have to be interpreted with some caution, and would benefit from a replication. Finally, we did not include several potentially relevant measures, such as maternal attunement and parent–infant synchrony, which particularly constraints the interpretation of our null findings.

In sum, we did not find support for the hypothesis that caregiver touch affects infant social orienting through the release of oxytocin. However, the link between oxytocin and social attention was replicated in our study. Given previous reports that kangaroo care affects infant oxytocin levels (Hardin et al., 2020; Vittner et al., 2017), it seems possible that certain types of tactile stimulation provided for long enough durations would be capable of influencing infant social orienting through their effects on oxytocin. Future research should further investigate the conditions necessary for touch to affect infant hormonal response. In particular, studies on parent–infant synchrony, neural response to touch, and interactions between tactile stimulation and infant past experiences with touch would be beneficial to our understanding of the impact of caregiver touch on infant development.

## CONFLICT OF INTEREST

The authors declare no conflict of interest.

## Supporting information

Supporting Information

## Data Availability

The data that support the findings of this study are available from the corresponding author upon reasonable request.
