# Supplementary material for: Oxytocin but not naturally occurring variation in caregiver touch associates with infant social orienting
Source: Dev Psychobiol. 2022 Jun 8;64(6):e22290. doi: 10.1002/dev.22290 (PMC9328151; doi:10.1002/dev.22290)
Supplement: Supplementary file 1 — Supporting Information [file DEV-64-0-s001.docx]

**Supplementary Material**

The analyses reported below were conducted in JASP (JASP Team, 2021).

1. Statistical analyses performed on the subset of pairwise-deleted complete cases

Section 2.2. Does caregiver touch predict infant oxytocin levels?

- - 1. Model predicting infant OT1 with infant age group and the PICTS score (F(2, 35) = 0.11, p = 0.90, R^2^ = 0.01): neither age group (β = 0.04, SE = 0.34, t = 0.12, p = 0.91) nor the PICTS (β = -0.07, SE = 0.17, t = -0.44, p = 0.66) were significant predictors.
    2. Model predicting change in infant oxytocin levels with age group and Observed Touch (F(2, 26) = 0.27, p = 0.76, R^2^ = 0.02): neither age group (β = 0.17, SE = 0.43, t = 0.39, p = 0.79) nor Observed Touch (β = 0.16, SE = 0.22, t = 0.74, p = 0.46) were significant predictors

Section 2.3. Does caregiver touch predict infant social attention?

2.3.1. Model predicting infant Face Pop Out scores with age group and the PICTS score (F(2, 58) = 0.70, p = 0.50, R^2^ = 0.02): neither age group (β = -0.23, SE = 0.26, t = -0.87, p = 0.39) nor the PICTS score were significant predictors.

2.3.2. Model predicting infant Face Pop Out scores with age group and Observed Touch (F(2, 58) = 1.02, p = 0.37, R^2^ = 0.03): neither age group (β = -0.36, SE = 0.28, t = -1.30, p = 0.20) nor Observed Touch (β = -0.16, SE = 0.15, t = -1.04, p = 0.30) were significant predictors.

Section 2.4. Does oxytocin predict infant social attention?

2.4.1. Model predicting infant Face Pop Out score with OT AUC (F(1, 25) = 4.45, p = 0.045, R^2^ = 0.15): OT AUC was a significant predictor (β = 0.43, SE = 0.20, t = 2.11, p = 0.045).

1. Comparison between infants with and without any oxytocin data in the main variables of interest

**Table S1.** Group means and differences in variables of interest between the infants who did and did not provide OT data points

As evidenced by the analyses below, no significant differences between infants with and without oxytocin data with regards to the main variables of interest were found.

**Predicting Face Pop Out with presence/lack of OT data and age group:**

| **Model Summary – Face Pop Out** | | | | | | | | | |
| --- | --- | --- | --- | --- | --- | --- | --- | --- | --- |
| **Model** | | **R** | | **R²** | | **Adjusted R²** | | **RMSE** | |
| H₀ |  | 0.000 |  | 0.000 |  | 0.000 |  | 0.157 |  |
| H₁ |  | 0.259 |  | 0.067 |  | 0.036 |  | 0.154 |  |
|  | | | | | | | | | |

| **ANOVA** | | | | | | | | | | | | | |
| --- | --- | --- | --- | --- | --- | --- | --- | --- | --- | --- | --- | --- | --- |
| **Model** | |  | | **Sum of Squares** | | **df** | | **Mean Square** | | **F** | | **p** | |
| H₁ |  | Regression |  | 0.105 |  | 2 |  | 0.052 |  | 2.189 |  | 0.121 |  |
|  |  | Residual |  | 1.456 |  | 61 |  | 0.024 |  |  |  |  |  |
|  |  | Total |  | 1.560 |  | 63 |  |  |  |  |  |  |  |
|  | | | | | | | | | | | | | |
|  | | | | | | | | | | | | | |

| **Coefficients** | | | | | | | | | | | | | |
| --- | --- | --- | --- | --- | --- | --- | --- | --- | --- | --- | --- | --- | --- |
| **Model** | |  | | **Unstandardized** | | **Standard Error** | | **Standardized** | | **t** | | **p** | |
| H₀ |  | (Intercept) |  | 0.479 |  | 0.020 |  |  |  | 24.358 |  | < .001 |  |
| H₁ |  | (Intercept) |  | 0.432 |  | 0.043 |  |  |  | 9.981 |  | < .001 |  |
|  |  | has_OT (1) |  | 0.081 |  | 0.043 |  |  |  | 1.886 |  | 0.064 |  |
|  |  | age_group (1) |  | -0.021 |  | 0.039 |  |  |  | -0.528 |  | 0.599 |  |
|  | | | | | | | | | | | | | |
|  | | | | | | | | | | | | | |

**Predicting Observed Touch with presence/lack of OT data and age group:**

| **Model Summary – Observed Touch** | | | | | | | | | |
| --- | --- | --- | --- | --- | --- | --- | --- | --- | --- |
| **Model** | | **R** | | **R²** | | **Adjusted R²** | | **RMSE** | |
| H₀ |  | 0.000 |  | 0.000 |  | 0.000 |  | 152.282 |  |
| H₁ |  | 0.403 |  | 0.163 |  | 0.137 |  | 141.478 |  |
|  | | | | | | | | | |

| **ANOVA** | | | | | | | | | | | | | |
| --- | --- | --- | --- | --- | --- | --- | --- | --- | --- | --- | --- | --- | --- |
| **Model** | |  | | **Sum of Squares** | | **df** | | **Mean Square** | | **F** | | **p** | |
| H₁ |  | Regression |  | 252673.626 |  | 2 |  | 126336.813 |  | 6.312 |  | 0.003 |  |
|  |  | Residual |  | 1.301e+6 |  | 65 |  | 20015.956 |  |  |  |  |  |
|  |  | Total |  | 1.554e+6 |  | 67 |  |  |  |  |  |  |  |
|  | | | | | | | | | | | | | |
|  | | | | | | | | | | | | | |

| **Coefficients** | | | | | | | | | | | | | |
| --- | --- | --- | --- | --- | --- | --- | --- | --- | --- | --- | --- | --- | --- |
| **Model** | |  | | **Unstandardized** | | **Standard Error** | | **Standardized** | | **t** | | **p** | |
| H₀ |  | (Intercept) |  | 269.424 |  | 18.467 |  |  |  | 14.590 |  | < .001 |  |
| H₁ |  | (Intercept) |  | 311.815 |  | 35.615 |  |  |  | 8.755 |  | < .001 |  |
|  |  | has_OT (1) |  | 17.675 |  | 36.960 |  |  |  | 0.478 |  | 0.634 |  |
|  |  | age_group (1) |  | -119.213 |  | 34.717 |  |  |  | -3.434 |  | 0.001 |  |
|  | | | | | | | | | | | | | |
|  | | | | | | | | | | | | | |

**Predicting PICTS with presence/lack of OT data and age group:**

| **Model Summary - PICTS_all** | | | | | | | | | |
| --- | --- | --- | --- | --- | --- | --- | --- | --- | --- |
| **Model** | | **R** | | **R²** | | **Adjusted R²** | | **RMSE** | |
| H₀ |  | 0.000 |  | 0.000 |  | 0.000 |  | 5.235 |  |
| H₁ |  | 0.133 |  | 0.018 |  | -0.013 |  | 5.268 |  |
|  | | | | | | | | | |

| **ANOVA** | | | | | | | | | | | | | |
| --- | --- | --- | --- | --- | --- | --- | --- | --- | --- | --- | --- | --- | --- |
| **Model** | |  | | **Sum of Squares** | | **df** | | **Mean Square** | | **F** | | **p** | |
| H₁ |  | Regression |  | 32.427 |  | 2 |  | 16.214 |  | 0.584 |  | 0.560 |  |
|  |  | Residual |  | 1803.789 |  | 65 |  | 27.751 |  |  |  |  |  |
|  |  | Total |  | 1836.217 |  | 67 |  |  |  |  |  |  |  |
|  | | | | | | | | | | | | | |

| **Coefficients** | | | | | | | | | | | | | |
| --- | --- | --- | --- | --- | --- | --- | --- | --- | --- | --- | --- | --- | --- |
| **Model** | |  | | **Unstandardized** | | **Standard Error** | | **Standardized** | | **t** | | **p** | |
| H₀ |  | (Intercept) |  | 54.272 |  | 0.635 |  |  |  | 85.488 |  | < .001 |  |
| H₁ |  | (Intercept) |  | 53.899 |  | 1.335 |  |  |  | 40.384 |  | < .001 |  |
|  |  | has_OT (1) |  | 1.084 |  | 1.391 |  |  |  | 0.779 |  | 0.439 |  |
|  |  | age_group (1) |  | -0.852 |  | 1.295 |  |  |  | -0.658 |  | 0.513 |  |
|  | | | | | | | | | | | | | |
|  | | | | | | | | | | | | | |

1. Associations between most commonly employed types of touch and difference in infant oxytocin levels and social orienting

We conducted a more fine-grained coding of caregiver touching behaviours based on ten descriptive categories (hug/hold, static, tickle, stroke/caress/move limbs/body, kiss/pat, touch with objects, rocking, games/routines played on body, massage). We coded the duration of each type of touching behaviour and added up the durations in PCI-FP and PCI-Q. The associations between the five most commonly employed types of touch and infant OT2-OT1 as well as Face Pop Out scores are shown in Table S1.

| Spearman's Correlations |  | | | | | |
| --- | --- | --- | --- | --- | --- | --- |
| Variable |  | **hugging/holding** | **stroking/caressing** | **moving limbs/body** | **kissing/patting** | **static** |
| OT2 - OT1 | Spearman's rho | 0.193 | -0.094 | 0.038 | -0.217 | -0.032 |
|  | p-value | 0.323 | 0.634 | 0.847 | 0.268 | 0.871 |
| Face Pop Out score | Spearman's rho | -0.003 | 0.104 | 0.078 | 0.089 | 0.026 |
|  | p-value | 0.984 | 0.432 | 0.555 | 0.504 | 0.843 |

**Table S2.** Spearman correlations between most commonly employed types of touch and infant OT2 – OT1 and Face Pop Out scores

1. Associations between proximity during free play and infant OT2-OT1 and Face Pop Out scores

Inspired by the results of a study by Krol and colleagues (2019), in which they found associations between maternal engagement and, in particular, the dimension of proximity, and methylation of the oxytocin receptor gene, we conducted additional analyses of our data. Five minutes of the free play interaction videos were coded for proximity on a Likert scale from 1 (very far) to 5 (very close). We were interested in whether we could observe a positive association between the proximity dimension and infant difference in oxytocin levels (OT2-OT1), oxytocin AUC and Face Pop Out score.


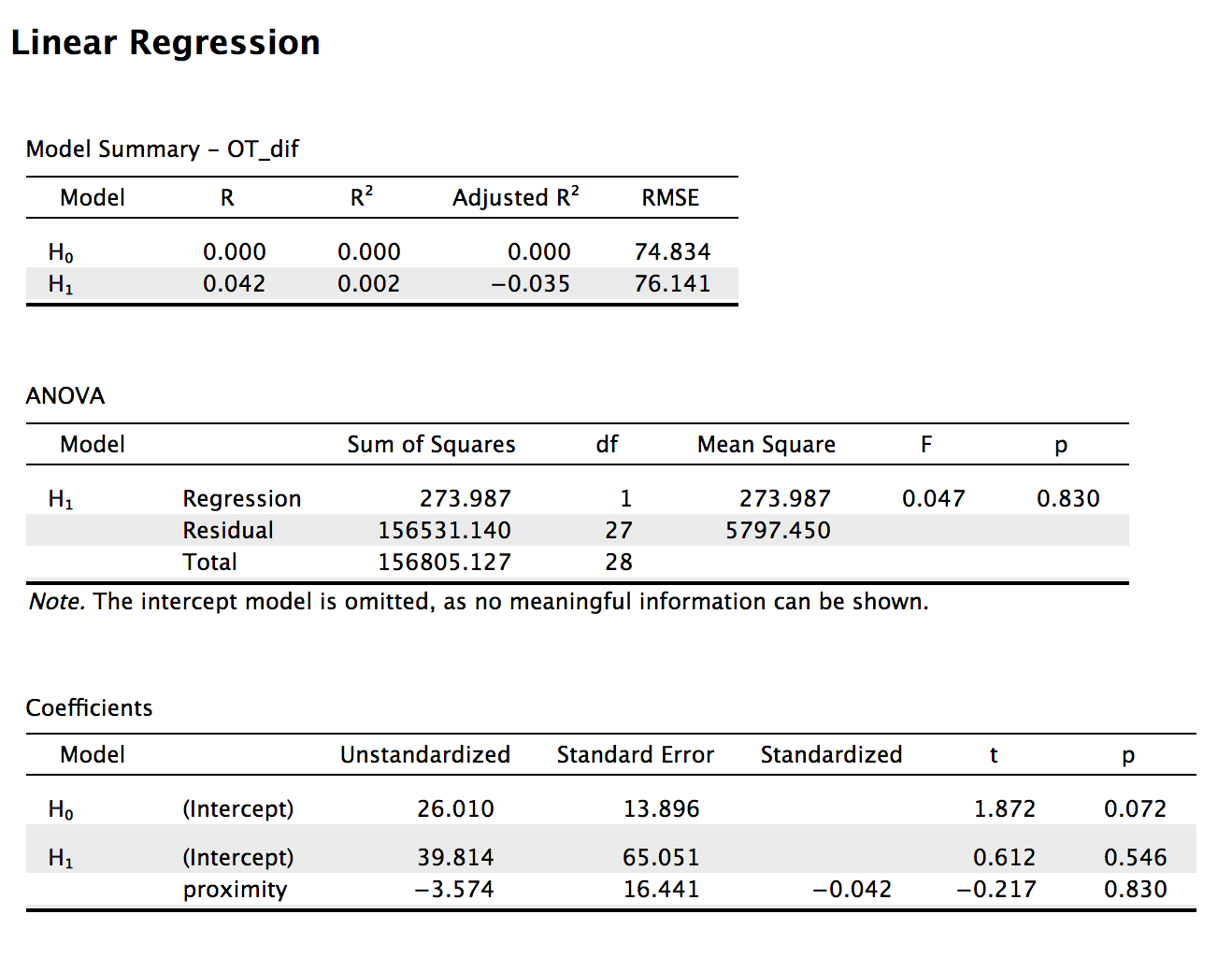
**Predicting OT2-OT1:**

No evidence that Proximity predicts OT2-OT1.

**
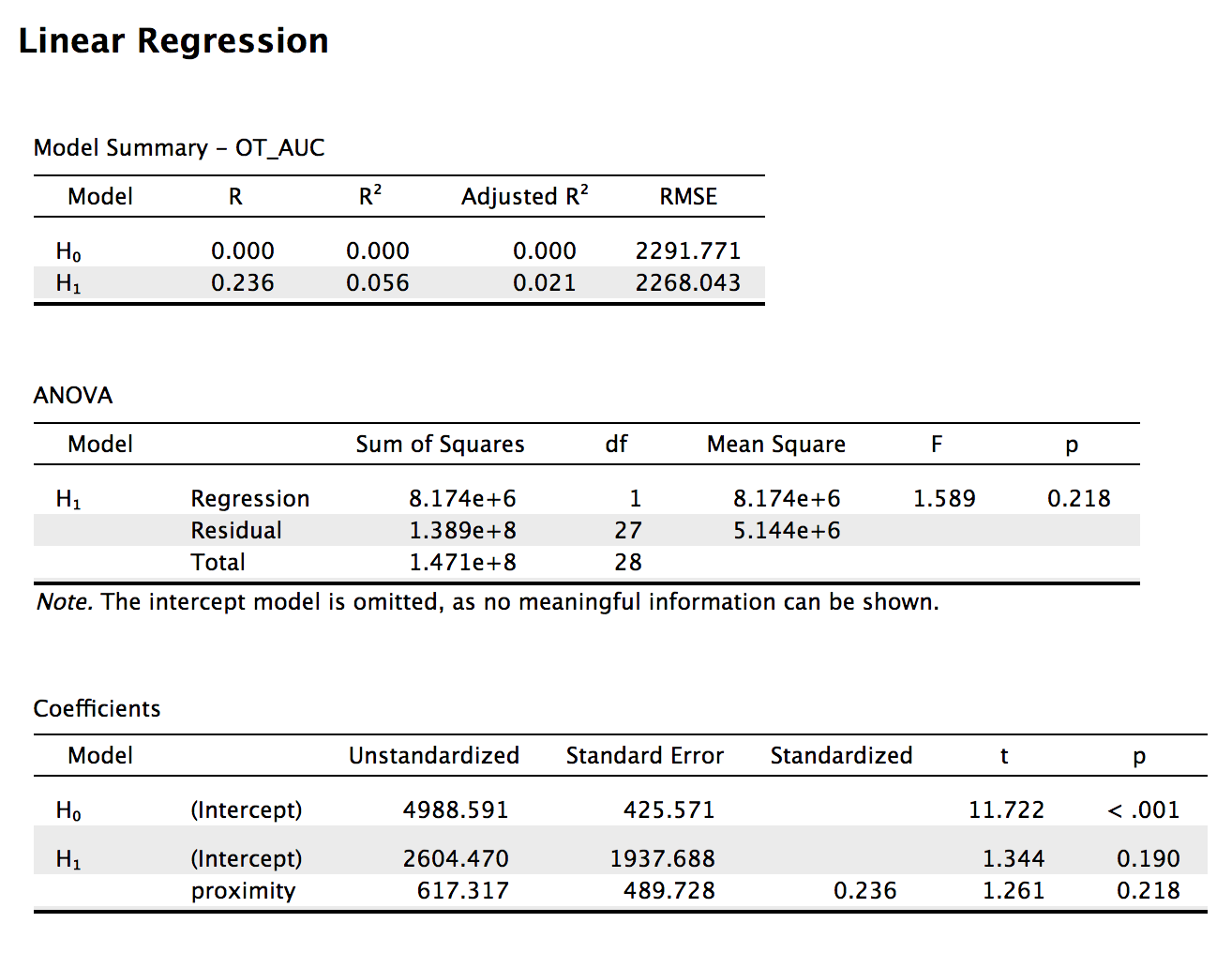
Predicting OT AUC:**

No evidence that Proximity predicts OT AUC.


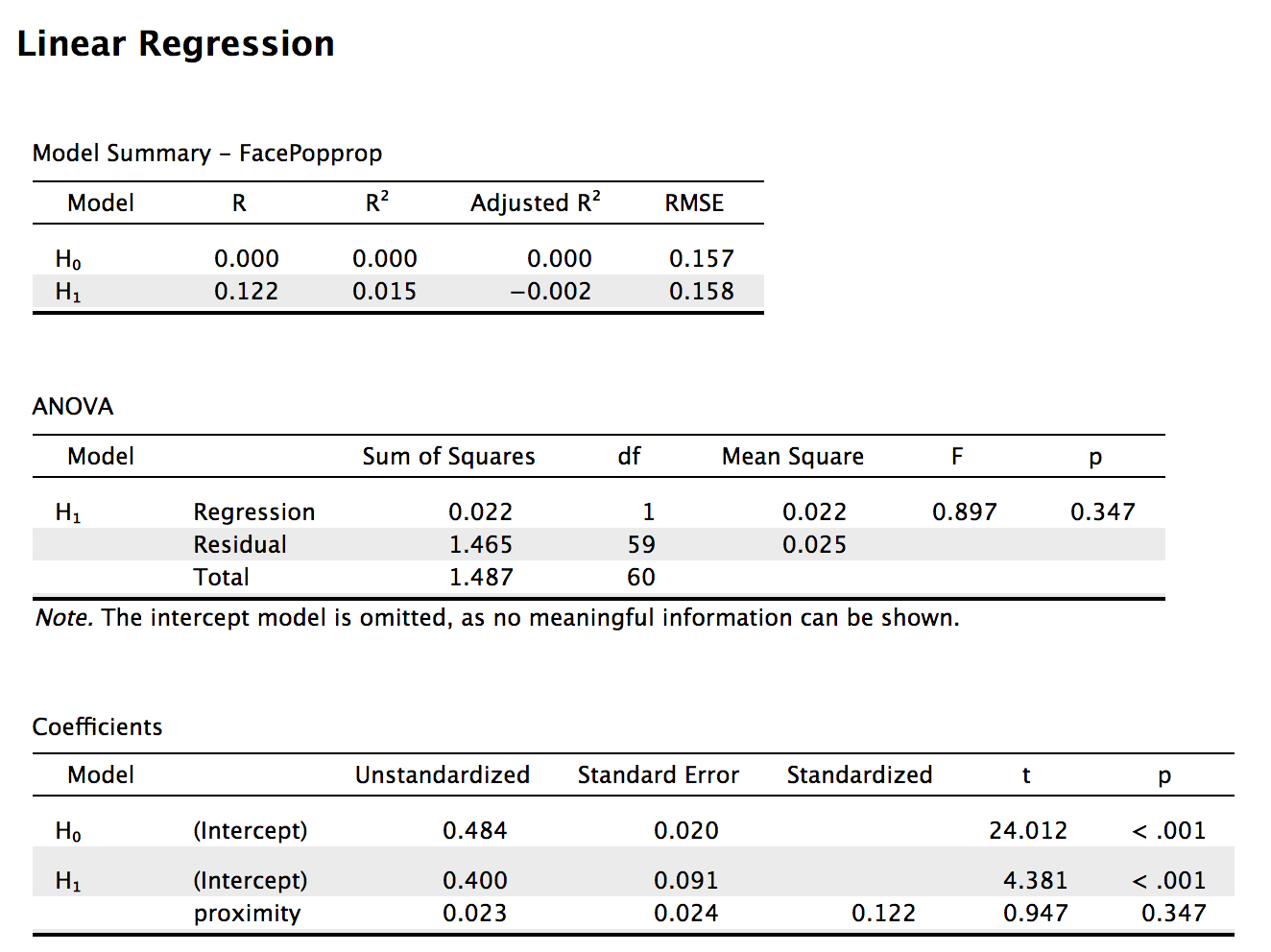
**Predicting Face Pop Out:**

No evidence that Proximity predicts Face Pop Out scores.

However, Proximity was positively correlated with the PICTS score, as well as the Observed Touch:


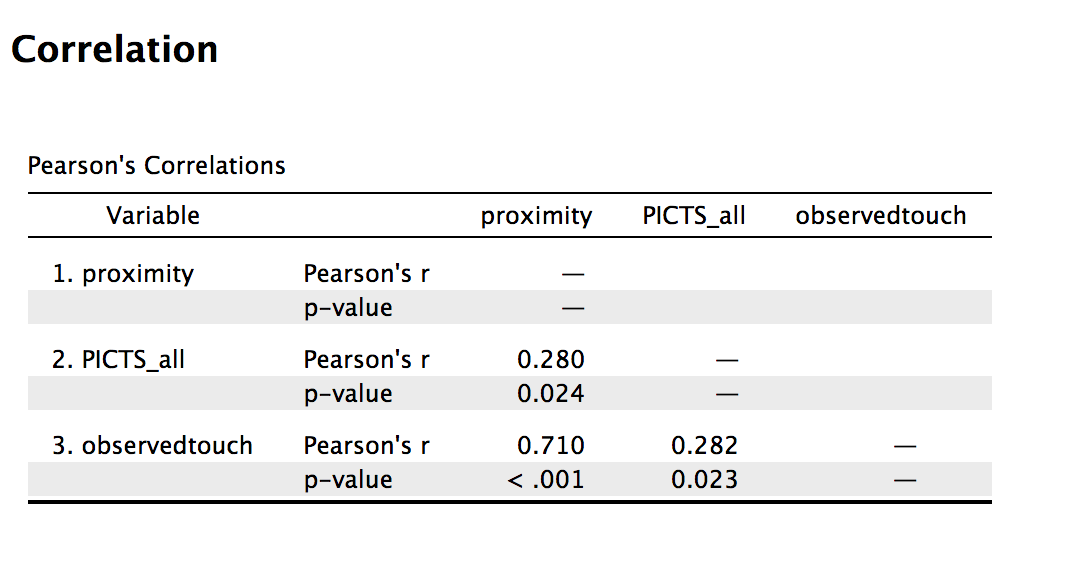


1. Associations between infant age and Face Pop Out scores

In the 6- to 8-month-olds group the mean was 0.50 (SD = 0.17), and in the 11-13-month-olds group it was 0.46 (SD = 0.14). The difference in Face Pop Out Proportion scores between the two age groups was not statistically significant (t(62) = 0.88, p = 0.38).


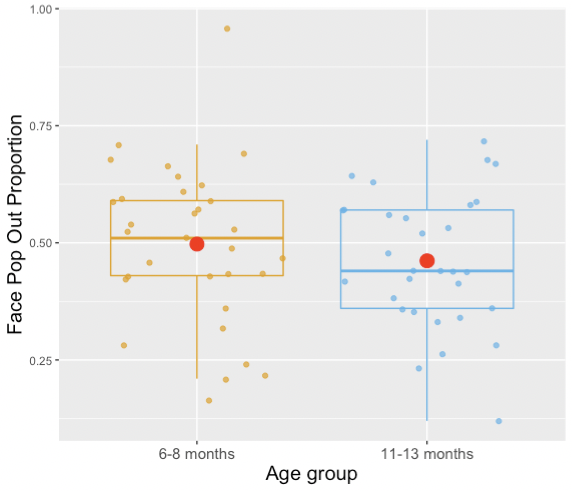


Figure S1. Boxplot showing the Face Pop Out Proportion scores in both age groups. All individual data are represented by points. Horizontal lines within boxplots indicate the median value, while red dots represent mean values.

We did not find an effect of infant age on either oxytocin or social attention in our study. Increased attention to faces presented among non-social objects develops around 6 months of age (Frank et al., 2014; Gliga et al., 2009); while the strong preference for social stimuli over non-social stimuli decreases over time (Hendry et al., 2018; Nishizato et al., 2017), our data suggests that this decrease might not happen until after 13 months of age.

**References**

Frank, M. C., Amso, D., & Johnson, S. P. (2014). Visual search and attention to faces during early infancy. *Journal of Experimental Child Psychology*, *118*(1), 13–26.

Gliga, T., Elsabbagh, M., Andravizou, A., & Johnson, M. (2009). Faces attract infants’ attention in complex displays. *Infancy*, *14*(5), 550–562. <https://doi.org/10.1080/15250000903144199>

Hendry, A., Jones, E. J. H., Bedford, R., Gliga, T., Charman, T., Johnson, M. H., Baron-Cohen, S., Blasi, A., Bolton, P., Cheung, H. M. C., Davies, K., Elsabbagh, M., Fernandes, J., Gammer, I., Green, J., Guiraud, J., Lloyd-Fox, S., Liew, M., Maris, H., … Wass, S. (2018). Developmental change in look durations predicts later effortful control in toddlers at familial risk for ASD. *Journal of Neurodevelopmental Disorders*, *10*(1). <https://doi.org/10.1186/s11689-017-9219-4>

JASP Team (2021). JASP (Version 0.16) [Computer software].

Nishizato, M., Fujisawa, T. X., Kosaka, H., & Tomoda, A. (2017). Developmental changes in social attention and oxytocin levels in infants and children. *Scientific Reports*, *7*(1), 1–10. https://doi.org/10.1038/s41598-017-02368-x
